# Cognitive Control and Weight Loss After Bariatric Surgery: the BARICO Study

**DOI:** 10.1007/s11695-023-06744-7

**Published:** 2023-07-21

**Authors:** Emma Custers, Debby Vreeken, Lisa-Katrin Kaufmann, Natalia Pujol-Gualdo, Marije Asbreuk, Maximilian Wiesmann, Esther Aarts, Eric J. Hazebroek, Amanda J. Kiliaan

**Affiliations:** 1 Department of Medical Imaging, Anatomy, Preclinical Imaging Centre, Radboud University Medical Center, Nijmegen, The Netherlands; 2 Department of Bariatric Surgery, Vitalys, Part of Rijnstate Hospital, Arnhem, The Netherlands; 3 Donders Institute for Brain, Cognition, and Behavior, Geert Grooteplein 21N, 6525 EZ, Nijmegen, The Netherlands; 4 Division of Human Nutrition and Health, Wageningen University, Wageningen, The Netherlands

**Keywords:** Obesity, Bariatric surgery, Weight loss, Cognitive control, fMRI

## Abstract

**Background and Objectives:**

Bariatric surgery (BS) is an effective treatment for obesity. However, some individuals experience insufficient weight loss after surgery. Therefore, we investigated whether cognitive control affects weight loss after Roux-en-Y gastric bypass (RYGB).

**Methods:**

Within this exploratory observational study, part of the BARICO study (BAriatric surgery Rijnstate and Radboudumc neuroImaging and Cognition in Obesity), participants aged between 35 and 55 years eligible for RYGB were included. Before and after BS, body weight, (delta) BMI and percentage total body weight loss (%TBWL) were determined. Additionally, at baseline, Stroop task-performance, -activation and -connectivity were assessed by a color-word paradigm task during functional neuroimaging to determine the ability of participants to inhibit cognitive interference.

**Results:**

Seventy-six participants were included, of whom 14 were excluded from fMRI analysis, leaving 62 participants. Participants were aged 45.0 ± 5.9 years with a mean pre-surgery BMI of 40.2 ± 3.3 kg/m^2^, and 86% were women. Mean decrease in BMI was 13.8 ± 2.5 kg/m^2^, and mean %TBWL was 34.9 ± 6.3% 1 year after BS. Stroop task performance did not correlate with (delta) BMI and %TBWL. The inferior parietal/middle occipital gyrus, inferior frontal gyrus, and supplementary motor cortex were involved in cognitive interference, although activity in these regions did not predict weight loss after surgery. Lastly, generalized psychophysiological interaction did not provide evidence for (delta) BMI- and %TBWL-dependent connectivity modulation.

**Discussion:**

Cognitive control did not predict weight loss after surgery. Future studies should focus on longer follow-up periods to understand the relation between cognitive control and weight loss.

**Trial Registration:**

NL7090 (https://www.clinicaltrialregister.nl/nl/trial/28949)

**Graphical Abstract:**

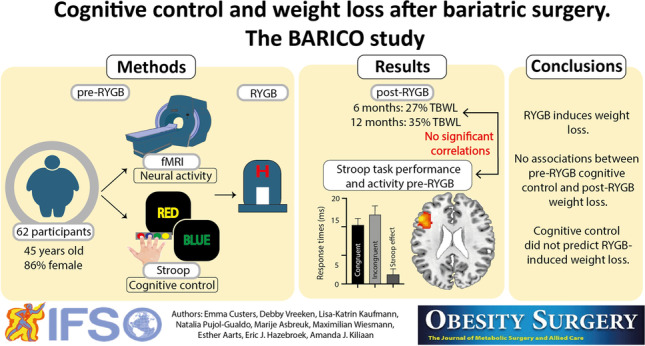

**Supplementary Information:**

The online version contains supplementary material available at 10.1007/s11695-023-06744-7.

## Introduction

Obesity and its comorbidities are major health problems of today’s society [1]. Bariatric surgery (BS), especially the commonly performed Roux-en-Y gastric bypass (RYGB), is an effective treatment for severe obesity leading to long-term weight loss, improvement of obesity-related comorbidities, increased quality of life, and finally, prolonged survival [2]. Patients undergoing RYGB lose on average 30% of their total body weight [3]. However, a large number of persons (25–30%) experience insufficient weight loss (defined as <25% total weight loss) or regain weight (defined as >25% of total lost weight after surgery) [4]. Weight regain tends to be a multifactorial problem where life style, socioeconomic status, demographics, metabolic imbalances, mental health, and surgical and anatomical factors might be involved [5]. In this paper, we are particularly interested in the role of cognitive control on weight loss after BS, as some patients have poor inhibitory control and cannot suppress the drive to eat when exposed to food stimuli [6].

Neural mechanisms of cognitive control are involved in initiating, coordinating, and updating behavior, collectively known as executive function [7]. Executive functioning promotes self-control and stimulates the pursuance of goal-oriented behaviors. In contrast, inadequate self-monitoring, sedentary behavior, and maladaptive eating behaviors are associated with executive dysfunction, which could result in higher body mass [7]. Furthermore, it has been demonstrated that regions involved in executive function, such as the prefrontal cortex (PFC) and the anterior cingulate cortex [8], are showing diminished activation in subjects with obesity while performing these tasks [9].

Executive function, particularly inhibitory control, can be experimentally measured with standardized validated tests, such as the color-word Stroop task [10]. During this task, participants are presented with color words written either in the same color as the word or in an incongruent color. Participants must indicate the color of the letters, ignoring the word’s meaning. Previous studies revealed that poorer Stroop performance is associated with higher body mass index (BMI) [11, 12]. Moreover, higher Stroop task reaction time interference (i.e., Stroop effect: incongruent-congruent) predicted weight gain in adults with a BMI ranging from 18 to 43 kg/m^2^ [12]. Additionally, functional near-infrared spectroscopy measurements performed during the Stroop task showed that subjects with obesity who had a high hemodynamic response in the bilateral dorsolateral and left ventrolateral PFC achieved more weight loss compared to subjects with lower neural activation in these regions [7]. Moreover, using a Stroop task with colored food and non-food words during fMRI, Janssen et al. demonstrated that obesity was associated with decreased dorsolateral PFC responses during attentional bias to food words as well as impaired goal-directed control during food choices [13]. Accordingly, it is suggested that inhibitory control deficits increase overeating and lead to poorer weight loss and maintenance after treatment [14].

In this study, we investigated the involvement of neurocognitive mechanisms in predicting post-surgical weight loss and maintenance. Sixty-two participants with severe obesity (average BMI of 40.3 kg/m^2^) from the BARICO study (BAriatric surgery Rijnstate and Radboudumc neuroImaging and Cognition in Obesity) performed the Stroop task during fMRI. We hypothesized that participants with higher Stroop interference control in the brain and behavior would more successfully lose weight compared to participants with worse interference control. To date, the reason for insufficient weight loss after BS is still largely unknown. Identifying and understanding if and how cognitive control is involved in insufficient weight loss after BS might improve clinical weight management interventions.

## Material and Methods

### Participants

Within this exploratory observational study, we analyzed data from participants of a subgroup of the BARICO study [15]. Between September 2018 and December 2020, 76 participants were recruited at the Rijnstate Hospital (Arnhem, the Netherlands), who underwent fMRI. Follow up finished in December 2021. Participants were between 35 and 55 years old at recruitment and selected for RYGB. Neurological or severe psychiatric illness, pregnancy, and treatment with antibiotics, probiotics or prebiotics 3 months before, or at any point during the study (excluding preoperative prophylaxis), claustrophobia, epilepsy, pacemakers and defibrillators, nerve stimulators, intracranial clips, infraorbital or intraocular metallic fragments, cochlear implants, ferromagnetic implants, shoulder width above the MRI space capacity, color blindness, and left handedness (to create a more homogenous sample with less variance) were exclusion criteria.

Participants performed a Stroop task during fMRI before surgery. Before, 6 months and 1 year after surgery, anthropometric data were recorded. Of these 76 participants, 14 were excluded, leaving 62 participants eligible for analysis (Figure S1).

### Standard Protocol Approvals, Registrations, and Patient Consents

The study was approved by the Medical Ethics Committee CMO region Arnhem–Nijmegen (NL63493.091.17) and by the local institutional ethics committee. The study was conducted in accordance with the ethical standards of the institutional research committee and with the Declaration of Helsinki “Ethical Principles for Medical Research Involving Human Subjects” as well as the guidelines for Good Clinical Practice (CPMP/ICH/135/95). Informed consent was obtained from all individual participants included in the study. The study was prospectively registered in the Netherlands Trial Registry (https://www.clinicaltrialregister.nl/nl/trial/28949)

### Medical Examination

Anthropometric measurements included body weight, BMI, delta BMI, and %TBWL. BMI was calculated as weight in kg divided by height in meters squared. Delta BMI was calculated 6 months and 1 year post-surgery. %TBWL was defined as weight loss divided by total weight before RYGB.

### Stroop Task

During the Stroop task, a color word is presented on the screen, and participants have to indicate the color of the letters by pressing the correct button reflecting that color, ignoring the meaning of the word. There are congruent color words, e.g., the word “RED” printed in red, and incongruent color words, e.g., the word “RED” printed in blue. The software of Presentation version 3.0 (Neurobehavioral Systems Inc. https://neurobs.com) was used to program and present the task. Before the MRI scan, participants were familiarized with the task. Participants were instructed to remember the correct color reflecting each button. Within the scanner, participants received a button box in their right hand, reflecting the four colors in which the word could be displayed (i.e., red, blue, yellow, or green). Button-color contingencies remained the same during the task and were counterbalanced across participants. The task stimuli were presented on a screen at the back end of the scanner bore, which was visible via a mirror fixed on the head coil. Within the scanner, 8 practice trials were performed with feedback (correct/incorrect). Each trial consisted of a jittered fixation period of 2–4 s followed by a colored word, which remained on the screen for 1.5 s. After the practice trials, participants were presented with 40 congruent and 40 incongruent trials. No feedback was given. The order of words was pseudorandomized and counterbalanced across participants.

Afterwards, the mean response time (RT) of correct responses to congruent and incongruent words was calculated, and error rates were determined. The Stroop effect was calculated as follows: mean RT to congruent words was subtracted from mean RT to incongruent words. The same was done for error rates. A higher interference score indicates less executive control ability.

### Imaging and fMRI Analyses

Participants were scanned in a 3T Skyra scanner (Siemens Healthineers, Erlangen, Germany) using a 32-channel head coil. Acquisition protocol included a 3D T1-weighted magnetization-prepared rapid gradient-echo sequence (TR/TI/TE 2300/1100/3.03 ms; 8° flip angle; voxel size: 1 mm isotropic) and a multiband, multi-echo planar imaging sequence to measure BOLD contrast during the Stroop task (TR/TE 1500/12.4, 34.3, 56.2 ms; 75° flip angle; voxel size: 2.5 × 2.5 × 2.5 mm; field of view 210 mm; 51 transversal slices in interleaved order). Data were preprocessed using fMRIPrep 22.0.1 [16], based on Nipype 1.8.4 [17].

#### Anatomical Data Preprocessing

T-1 weighted (T1w) images were corrected for intensity non-uniformity with N4BiasFieldCorrection [18], distributed with ANTs 2.3.3 [19], and used as T1w-reference. T1w-reference was skull-stripped with a Nipype implementation of the antsBrainExtraction.sh workflow (ANTs), with OASIS30ANTs as template. Brain tissue segmentation was performed on the skull-stripped T1w-reference using FAST (FSL 6.0.5.1:57b01774) [20]. With recon-all, brain surfaces were reconstructed (FreeSurfer 7.2.0) [21]. With nonlinear registration (ANTs), volume-based spatial normalization was performed. For spatial normalization, the FSL’s MNI ICBM 152 non-linear 6th Generation Asymmetric Average Brain Stereotaxic Registration Model [22] template was selected using a resolution of 2-mm isotropic voxels.

#### Functional Data Pre processing

The shortest echo of the BOLD images was used to generate a reference volume and its skull-stripped version using a custom methodology of fMRIprep. Before any spatiotemporal filtering, head-motion parameters with respect to the BOLD reference (transformation matrices and six corresponding rotation and translation parameters) were estimated using MCFLIRT (FSL6.0.5.1:57b01774) [23]. BOLD images were slice-time corrected to 0.696 s using 3dTshift from analysis of functional neuroimages (AFNI) [24]. Slice-time corrected-BOLD-time series were resampled onto their original, native space by applying the transforms. A T2* map was estimated from the preprocessed EPI echoes by voxel-wise fitting the maximal number of echoes with reliable signal in that voxel to a mono-exponential signal decay model with nonlinear regression. Calculated T2* map was then used to optimally combine resampled BOLD-time series across echoes following the method described elsewhere [25]. For a more detailed description of fMRIPrep see supplementary material.

Further processing and analyses were performed with SPM12 (v7771; Wellcome Center for Neuroimaging, London, UK; www.fil.ion.ucl.ac.uk/spm/software/spm12). After removing the first 30 dummy scans per echo, images were smoothed with a Gaussian kernel of 8 mm full width at half maximum.

Statistical analysis was performed using a general linear model (GLM) approach. At the first level, subject-specific data was analyzed in an event-related design by a fixed effects model, which included two regressors of interest that reflected the onset of congruent and incongruent words, plus nuisance regressors for CSF and WM, and motion artifacts derived from independent component analysis [26]. Trials were modeled with a stick function aligned to the onset of each stimulus, convolved with the canonical hemodynamic response function. Serial correlations in the time series were accounted for using a first-degree autoregressive model, and low-frequency drifts were removed using a high-pass filter at 1/128 Hz. A second-level analysis was performed to investigate whole-brain group effects. The main Stroop task effect was analyzed using a one-sample *t*-test on the incongruent > congruent contrast across all participants. To correct for multiple comparisons over the whole brain, a cluster-level threshold of pFWE < 0.05 was used (clusters identified at *p* < 0.001 uncorrected). The mean betas were extracted from the three largest clusters within the whole-brain search volume. These betas were used for further linear regression analyses.

Additionally, we performed a generalized psychophysiological interaction (gPPI) analysis [27] to test whether functional connectivity is associated with (delta) BMI and %TBWL. As seeds, we used the three largest clusters that showed greater activity for the incongruent > congruent contrast. Two search volumes were used: one limited to areas involved in generally executive function tasks [28], and one covering the whole brain gray matter (using the tissue probability map binarized at 30% tissue probability). Multiple regression models were calculated for each target voxel of the brain, comprising the following independent variables: [1] the two task conditions convolved with the canonical hemodynamic response function; [2] the eigenvariate of the seed time series; [3] the interaction terms specified as products of [1] and [2] (PPI terms); and additional regressors for age, sex, and pre-surgery BMI. Statistical inference was performed at the cluster level, with a cluster-defining threshold of *p* < 0.001, controlling the family-wise-error rate at *α* ≤ 0.05 across the search volume. Since the gPPI analysis models the main effect of the task, gPPI only detects functional connectivity effects over and above the main task effect.

### Analyses of Behavioral Data

Mean RT, error rates, and interference scores were calculated. To ensure proper task execution, participants with less than 50% accuracy in both congruent and incongruent trials were excluded from further analyses. Using the Pearson correlation coefficient, the relationship with (delta) BMI and %TBWL was investigated.

Statistical analyses were performed using IBM SPSS Statistics version 27 (SPSS Inc., Chicago, IL). Normality was checked for all continuous variables. When assumptions on normality were not met, a natural log transformation or nonparametric test was used. To test over time changes in patient characteristics, repeated measures analyses of variance (ANOVA) with Bonferroni correction were conducted.

With Pearson correlations (2-sided), we assessed the association between the Stroop task performance, (delta) BMI, and %TBWL. The relation between incongruent > congruent contrast in several brain regions and (delta) BMI and %TBWL was determined with (multivariable) linear regression models. Effects of confounding variables on the associations were explored using 3 models: no adjustment (crude model), adjustments for age and sex (model 2), and a full model with adjustments for age, sex, and pre-surgery BMI. Since this is an exploratory study, we did not correct for multiple comparisons. Alpha was set at 0.05 (2-tailed) for the descriptive analyses, Pearson correlations, and linear regression models.

## Results

### Descriptive Statistics

Participants characteristics are listed in Table 1; mean age of the participants was 45.0 ± 5.9 years with a mean BMI of 40.3 ± 3.3 kg/m^2^. Eighty-six percent of the study population were women. At 6 months after surgery, average weight and BMI significantly reduced, and even more 1 year after surgery (Table 1). Mean percentage of %TBWL 1 year after surgery was 34.9 ± 6.3%, and only 4.8% did not show sufficient weight loss 1 year after BS.

**Table 1:** Characteristics of participants (*n* = 62)

	Baseline	6 months	1 year	*p*-value
Age, mean ± SD, y	45.0 ± 5.9			n/a
Sex, women, *n* (%)	53 (86%)			n/a
Weight, mean ± SD, kg	116.6 ± 13.2	85.0 ± 11.1	77.0 ± 11.3	<0.001
BMI, mean ± SD, kg/m^2^	40.3 ± 3.3	29.5 ± 3.6	26.7 ± 3.6	<0.001
Delta BMI, mean ± SD, kg/m^2^		10.9 ± 2.3	13.8 ± 2.5	< 0.001
TBWL, mean ± SD, %		27.1 ± 5.6	34.9 ± 6.3	<0.001
Level of education^a^, *n* (%)				
Low	7 (11%)			n/a
Middle	31 (50%)			n/a
High	24 (39%)			n/a

Repeated measures analyses of variance were conducted to examine changes in characteristics over time. Significant changes over time are indicated by underscoring the *p*-values

^a^A Verhage score ≤4 is defined as low level of education, a Verhage score of 5 as middle, a Verhage score of 6 or 7 as high level of education [29]

Abbreviations: *n/a* not applicable, *BMI* body mass index, *TBWL* total body weight loss

### Stroop Task Performance

RT and error rates for congruent and incongruent words, and the Stroop effect are presented in Table 2.

**Table 2:** Response times and error rates of the Stroop task

Stroop task performance			
	Congruent	Incongruent	Stroop effect
Response time, mean ± SD, ms	827.28 ± 101.23	985.03 ± 126.58	130.76 ± 86.74
Error rate, median (IQR), %	2.50 (5.0)	7.50 (10.0)	5.00 (10.0)

With Pearson correlations, we assessed correlations between the Stroop task performance (RT and error rate) and (delta) BMI and %TBWL at different time points (Table 3, Table S1). No significant correlations were found (smallest *p*-value = 0.24).

**Table 3:** Correlation coefficients between Stroop performance and weight loss

	Delta BMI		%TBWL	
	6 months	1 year	6 months	1 year
Response times				
Congruent	0.09	0.01	0.10	0.03
Incongruent	0.15	0.06	0.12	0.05
Stroop effect	0.11	0.07	0.07	0.04
Error rate				
Congruent	0.02	0.11	0.05	0.13
Incongruent	−0.01	−0.02	−0.03	−0.03
Stroop effect	−0.02	−0.06	−0.04	−0.07

The Pearson’s (*R*) test was used

Abbreviations: *BMI* body mass index, *TBWL* total body weight loss

### Stroop Task Activation

**Fig. 1:**
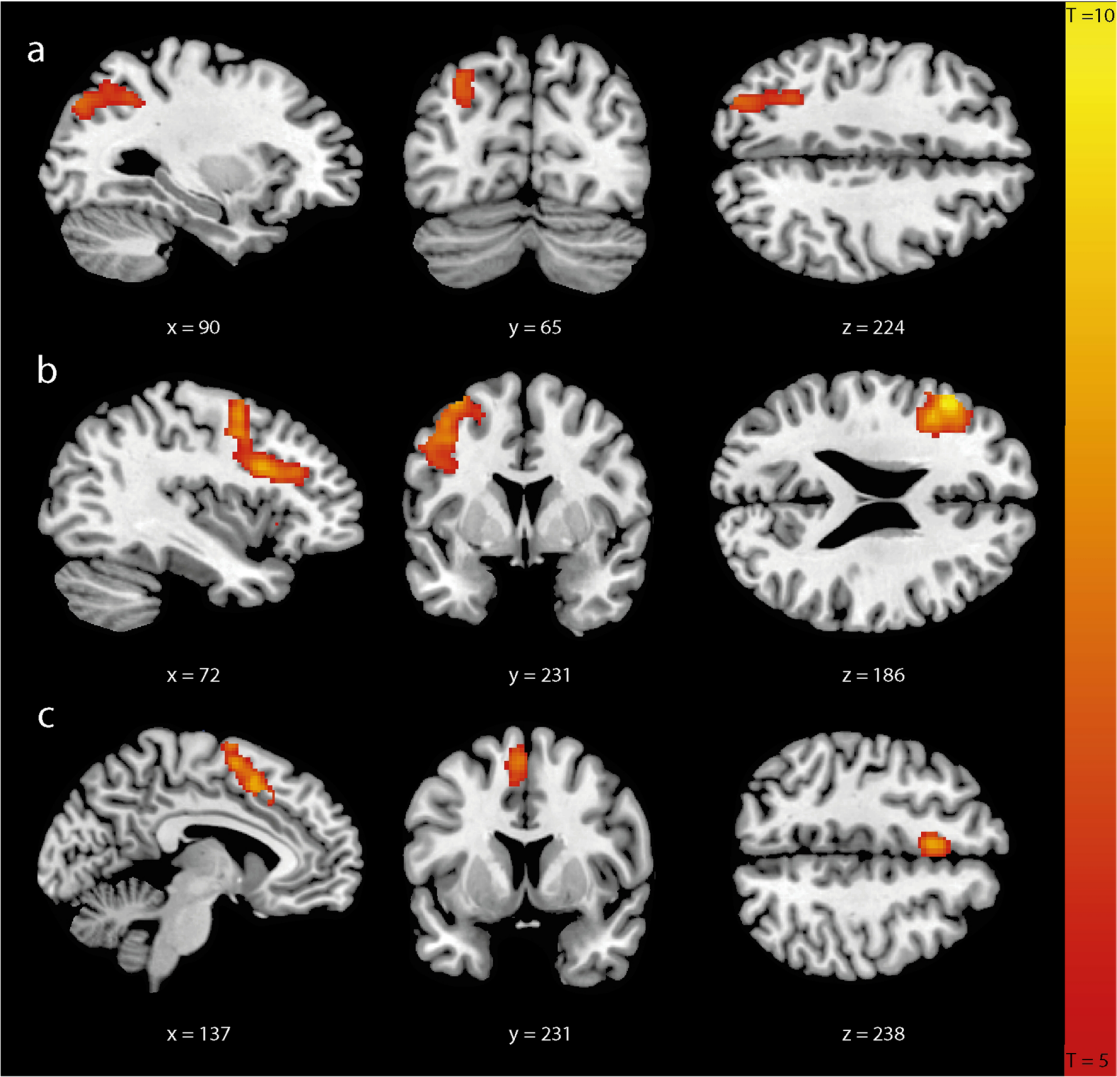
Neural Stroop interference effect. Contrast of incongruent versus congruent words (incongruent > congruent) in the inferior parietal/middle occipital gyrus (**a**); inferior frontal gyrus (**b**); and supplementary motor cortex (**c**). Images are shown in neurological convention (left = left) with sagittal, coronal, and axial slice coordinates as defined in MNI152 space. For illustrative purposes full brain statistical parametric maps were threshold at pFWE (cluster) < 0.05, and corrected for age and sex

We investigated neural networks involved in the attentional bias to incongruent and congruent words. When contrasting activation in response to incongruent relative to congruent (incongruent-congruent) words, we found a main effect in several clusters throughout the brain. Among these, the inferior parietal/middle occipital gyrus, inferior frontal gyrus, and supplementary motor cortex were the largest significant clusters (Fig. 1, Figure S2, Figure S3).

To determine whether Stroop-related brain responses can predict weight loss over time, we extracted the beta weights of the incongruent > congruent contrast of the inferior parietal/middle occipital gyrus, inferior frontal gyrus, and supplementary motor cortex and used these in linear regression analysis. For all three regions, no significant correlations were found with (delta) BMI and %TBWL (Table 4, Table S2).

**Table 4:** Linear regression models estimating the relation between brain activation during response inhibition and weight loss

Beta clusters			
	Inferior parietal/middle occipital gyrus	Inferior frontal gyrus	Supplementary motor cortex
	*β* (95% CI)	*β* (95% CI)	*β* (95% CI)
Delta BMI 6 months			
Crude model	0.04 (−0.23 to 0.31)	−0.11 (−0.37 to 0.15)	0.02 (−0.24 to 0.28)
Adjusted for age and sex	0.07 (−0.20 to 0.34)	−0.09 (−0.35 to 0.17)	0.02 (−0.24 to 0.28)
Full model	0.04 (−0.23 to 0.32)	−0.10 (−0.35 to 0.16)	0.02 (−0.24 to 0.28)
Delta BMI 1 year			
Crude model	0.04 (−0.23 to 0.31)	−0.12 (−0.68 to 0.14)	0.07 (−0.19 to 0.33)
Adjusted for age and sex	0.03 (−0.24 to 0.29)	−0.11 (−0.36 to 0.15)	0.07 (−0.18 to 0.33)
Full model	0.00 (−0.26 to 0.26)	−0.12 (−0.37 to 0.14)	0.07 (−0.18 to 0.32)
%TBWL 6 months			
Crude model	−0.02 (−0.29 to 0.25)	−0.13 (−0.38 to 0.13)	0.02 (−0.24 to 0.28)
Adjusted for age and sex	−0.01 (−0.28 to 0.27)	−0.11 (−0.37 to 0.15)	0.02 (−0.24 to 0.29)
Full model	−0.04 (−0.23 to 0.32)	−0.10 (−0.36 to 0.16)	−0.15 (−0.24 to 0.27)
%TBWL 1 year			
Crude model	−0.01 (−0.29 to 0.26)	−0.13 (−0.39 to 0.13)	0.08 (−0.18 to 0.34)
Adjusted for age and sex	−0.01 (−0.29 to 0.26)	−0.13 (−0.38 to 0.14)	0.08 (−0.18 to 0.33)
Full model	0.00 (−0.27 to 0.28)	−0.12 (−0.38 to 0.14)	0.08 (−0.18 to 0.33)

Beta-coefficients with 95% confidence intervals. In the full model we adjusted for age, sex, and pre-surgery BMI

Abbreviations: *BMI* body mass index, *TBWL* total body weight loss

### Stroop Task Connectivity

The three largest clusters showing greater activity for the (incongruent > congruent) GLM contrast served as seed regions for the gPPI connectivity analyses. However, no evidence for connectivity modulation nor (delta) BMI- and %TBWL-dependent connectivity modulation during the task was observed for these regions at pFWE <0.05. Subthreshold changes of the task-dependent connectivity are depicted in Figure S4.

## Discussion

In this exploratory study, we aimed to investigate the effect of cognitive control on weight loss after BS. More specifically, we determined whether brain activity and connectivity during the Stroop task were associated with (delta) BMI and %TBWL after RYGB. Our findings suggest that cognitive control is not associated with pre- and post-surgery BMI, change in BMI, and %TBWL. We found no correlations between the Stroop effect, (delta) BMI, and %TBWL, nor associations between BOLD responses in the occipital and frontal cortex and (delta) BMI and %TBWL after BS. Additionally, no evidence was found for (delta) BMI- and %TBWL- dependent connectivity modulation during the Stroop task.

Our neural effects are not consistent with previous literature showing that high response inhibition predicts weight loss [12, 30–32]. In a cohort of 23 individuals with obesity who participated in a weight loss program, good performance on a set-shifting task and slower RT on a response inhibition test were associated with higher weight loss after 8 weeks [30]. In contrast to that study, our sample consists of a rather large patient group with a follow-up period of 12 months, perhaps expecting larger effects. However, compared to BS, weight loss through diet might depend more on cognitive control, possibly explaining these different results. Other evidence does show that baseline fMRI responses in the nucleus accumbens and ventromedial PFC/orbitofrontal cortex in response to passive viewing of palatable food stimuli can predict weight loss after sleeve gastrectomy [32]. Therefore, it is assumed that the neurobiology of behavioral changes supports %TBWL [31]. Unlike these previous reports focusing on food cues, we could not confirm that functional activity or connectivity during Stroop interference/inhibitory control is associated with or modulated by (delta) BMI and %TBWL. However, it should be noted that neurocognitive processing of food cues can also reflect reward sensitivity rather than executive function. Current and present findings might indicate that pre-surgery neurocognitive processing of food cues is more predictive for surgery-induced weight loss.

This study has limitations, which might also explain the lack of findings. In the current sample, almost all participants achieved good sustainable weight loss; only 4.8% did show insufficient weight loss after 1 year. This might induce the effect of selection bias. The BARICO study is an extensive study, with a long follow-up period; therefore, participants needed to be highly motivated. This selection bias might have led to the high percentage of successful weight loss in our study compared to the average bariatric population. Another limitation is the length of the follow-up period. We investigated whether behavioral and fMRI Stroop effects could predict weight loss 6 months or 1 year after surgery. Although BS is very effective during the first year, literature shows that 2 years post-surgery approximately 20% show insufficient weight loss [33, 34], indicating that we might be too early to identify cognitive control as a predictor for weight loss. In this study, no control group was included; therefore, it is difficult to determine if our cohort had a worse Stroop task performance compared to lean participants. Although, compared to other studies that focused on the Stroop effect in subjects with obesity [7, 35], we observed similar RTs. Lastly, it is important to keep the complexity of the mechanisms underlying weight loss and weight regain in mind. Not only cognitive control can contribute to weight loss, but also genetic factors, hormone levels, age, diet, physical activity, and mood might be involved [36, 37].

For future follow-up studies, it would be interesting to measure post-surgery cognitive control in relation to post-surgery weight loss. In addition, it is important to assess the post-surgery Stroop effect and compare this with the Stroop effect of patients with obesity who did not undergo BS. This would allow researchers to investigate whether the surgery has any effect on cognitive performance.

In conclusion, we found no associations between pre-surgery cognitive control and weight loss after BS. Thus, in this explorative study, cognitive control did not predict weight loss after surgery. Nevertheless, it is very important to unravel predictors for weight loss after BS to identify individuals who benefit the most from the intervention. By establishing pre-operative predictors of surgical outcomes, patient counseling regarding inadequate weight loss or weight regain can be improved.

## Supplementary Information

ESM 1

## Data Availability

Data will be available upon request after the BARICO study is finished.
