# Supplementary material for: Cognitive Control and Weight Loss After Bariatric Surgery: the BARICO Study
Source: Obes Surg. 2023 Jul 21;33(9):2799–807. doi: 10.1007/s11695-023-06744-7 (PMC10435598; doi:10.1007/s11695-023-06744-7)
Supplement: Supplementary file 1 — ESM 1 [file 11695_2023_6744_MOESM1_ESM.docx]

**Supplementary material**


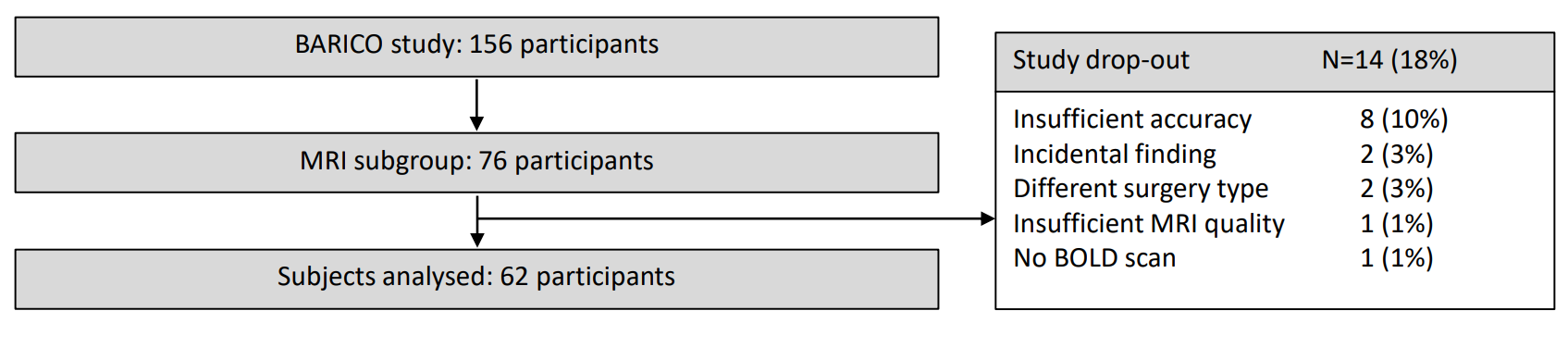


**Figure S1: Flowchart with included participants.** Of the 76 participants enrolled in the MRI group, eight participants were excluded due to insufficient accuracy during the task. Another two participants had to be excluded due to clinically relevant findings in the MRI (meningioma), two participants were excluded due to a last minute change of surgery (sleeve gastrectomy), one participant was excluded due to insufficient MRI quality (temporal lobe cut-off), and one participant had no BOLD scan due to time issues, leading to a total of 62 participants eligible for fMRI analyses.

**Supplementary imaging and fMRI preprocessing analyses**

Results included in this manuscript come from preprocessing performed using fMRIPrep 22.0.1 ^1^, which is based on Nipype 1.8.4 ^2^.

**Anatomical data preprocessing**

A total of 1 T1-weighted (T1w) images were found within the input BIDS dataset. The T1-weighted (T1w) image was corrected for intensity non-uniformity (INU) with N4BiasFieldCorrection ^3^, distributed with ANTs 2.3.3 ^4^, and used as T1w-reference throughout the workflow. The T1w-reference was then skull-stripped with a Nipype implementation of the antsBrainExtraction.sh workflow (from ANTs), using OASIS30ANTs as target template. Brain tissue segmentation of cerebrospinal fluid (CSF), white-matter (WM) and gray-matter (GM) was performed on the brain-extracted T1w using fast (FSL 6.0.5.1:57b01774)^5^. Brain surfaces were reconstructed using recon-all (FreeSurfer 7.2.0) ^6^, and the brain mask estimated previously was refined with a custom variation of the method to reconcile ANTs-derived and FreeSurfer-derived segmentations of the cortical gray-matter of Mindboggle ^7^. Volume-based spatial normalization to standard space (MNI152NLin6Asym), was performed through nonlinear registration with antsRegistration (ANTs 2.3.3), using brain-extracted versions of both T1w reference and the T1w template. The following templates were selected for spatial normalization: FSL’s MNI ICBM 152 non-linear 6th Generation Asymmetric Average Brain Stereotaxic Registration Model ^8^ [TemplateFlow ID: MNI152NLin6Asym].

**Functional data preprocessing**

For each of the 1 BOLD runs found per subject (across all tasks and sessions), the following preprocessing was performed. First, a reference volume and its skull-stripped version were generated from the shortest echo of the BOLD run using a custom methodology of fMRIPrep. Head-motion parameters with respect to the BOLD reference (transformation matrices, and six corresponding rotation and translation parameters) are estimated before any spatiotemporal filtering using mcflirt (FSL 6.0.5.1:57b01774) ^9^. BOLD runs were slice-time corrected to 0.696s (0.5 of slice acquisition range 0s-1.39s) using 3dTshift from AFNI ^10^. The BOLD time-series (including slice-timing correction when applied) were resampled onto their original, native space by applying the transforms to correct for head-motion. These resampled BOLD time-series will be referred to as preprocessed BOLD in original space, or just preprocessed BOLD. A T2* map was estimated from the preprocessed EPI echoes, by voxel-wise fitting the maximal number of echoes with reliable signal in that voxel to a monoexponential signal decay model with nonlinear regression. The T2*/S0 estimates from a log-linear regression fit were used for initial values. The calculated T2* map was then used to optimally combine preprocessed BOLD across echoes following the method described in ^11^. The optimally combined time series was carried forward as the preprocessed BOLD. The BOLD reference was then co-registered to the T1w reference using bbregister (FreeSurfer) which implements boundary-based registration ^12^. Co-registration was configured with six degrees of freedom. First, a reference volume and its skull-stripped version were generated using a custom methodology of fMRIPrep. The three global signals are extracted within the CSF and the WM. The BOLD time-series were resampled into standard space, generating a preprocessed BOLD run in MNI152NLin6Asym space. First, a reference volume and its skull-stripped version were generated using a custom methodology of fMRIPrep. Automatic removal of motion artifacts using independent component analysis (ICA-AROMA) ^17^ was performed on the preprocessed BOLD on MNI space time-series after removal of non-steady state volumes and spatial smoothing with an isotropic, Gaussian kernel of 6mm FWHM (full-width half-maximum). The “aggressive” noise-regressors were collected and placed in the corresponding confounds file. All resamplings can be performed with a single interpolation step by composing all the pertinent transformations (i.e. head-motion transform matrices, susceptibility distortion correction when available, and co-registrations to anatomical and output spaces). Gridded (volumetric) resamplings were performed using antsApplyTransforms (ANTs), configured with Lanczos interpolation to minimize the smoothing effects of other kernels^18^. Non-gridded (surface) resamplings were performed using mri_vol2surf (FreeSurfer).

Many internal operations of fMRIPrep use Nilearn 0.9.1 ^13^, mostly within the functional processing workflow. For more details of the pipeline, see the section corresponding to workflows in fMRIPrep’s documentation.

**Table S1.** Correlation coefficients between Stroop performance and BMI.

|  | BMI | | |
| --- | --- | --- | --- |
|  | Baseline | 6 months | 1 year |
| Response times |  |  |  |
| *Congruent* | -0.04 | -0.10 | -0.05 |
| *Incongruent* | 0.04 | -0.07 | -0.02 |
| *Stroop effect* | 0.10 | 0.02 | 0.02 |
| Error rate |  |  |  |
| *Congruent* | 0.09 | 0.10 | 0.15 |
| *Incongruent* | 0.00 | -0.01 | -0.01 |
| *Stroop effect* | 0.03 | 0.04 | 0.06 |

*The Pearson’s (R) test was used.*

*Abbreviations: BMI = body mass index, TBWL = total body weight loss.*


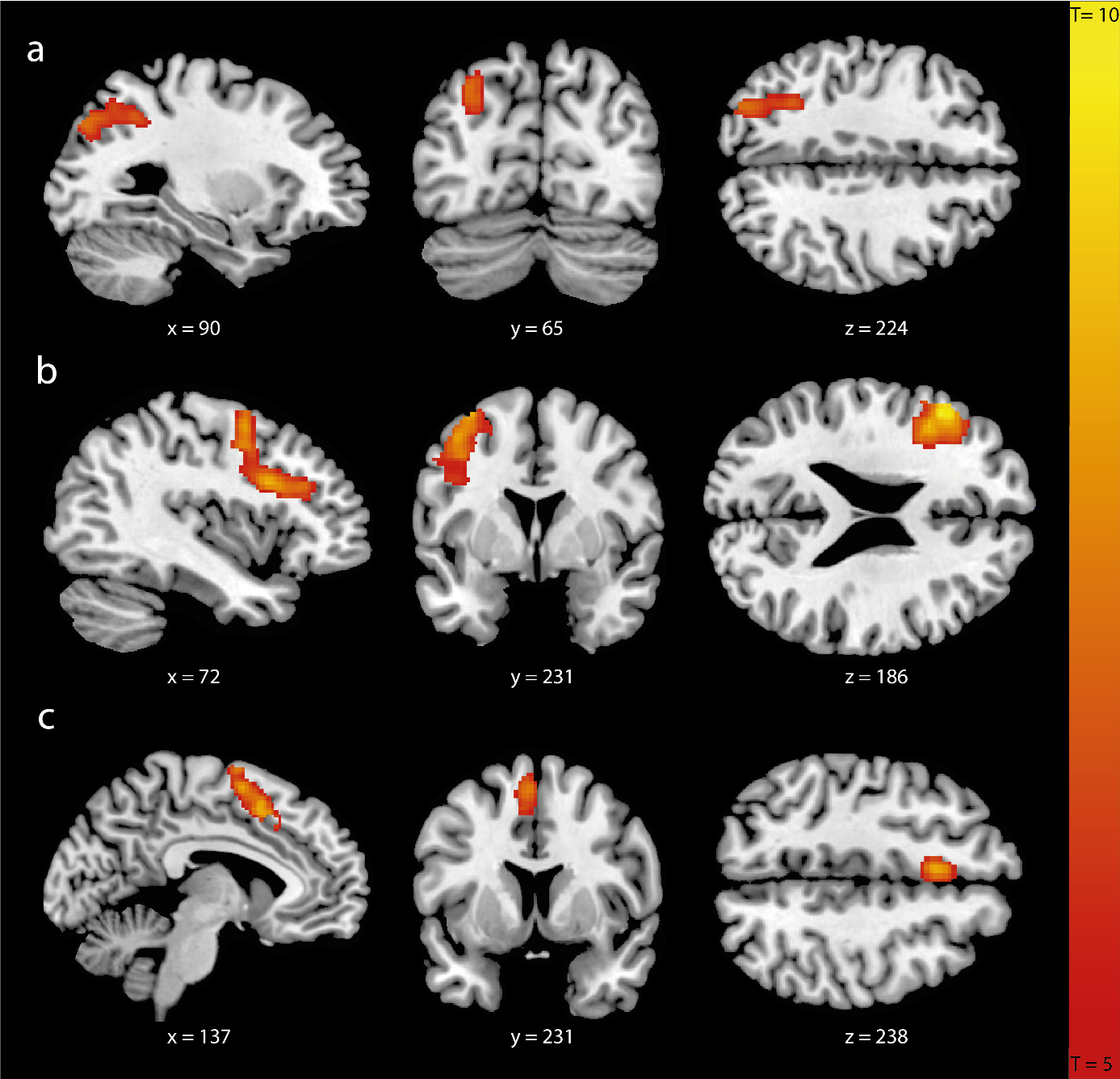


**Figure S2. Neural Stroop interference effect**. Contrast of incongruent versus congruent words (incongruent > congruent) in **a**) inferior parietal/middle occipital gyrus; **b**) inferior frontal gyrus; **c**) supplementary motor cortex. Images are shown in neurological convention (left=left) with sagittal, coronal and axial slice coordinates as defined in MNI152 space. For illustrative purposes full brain statistical parametric maps were thresholded at pFWE < 0.05.


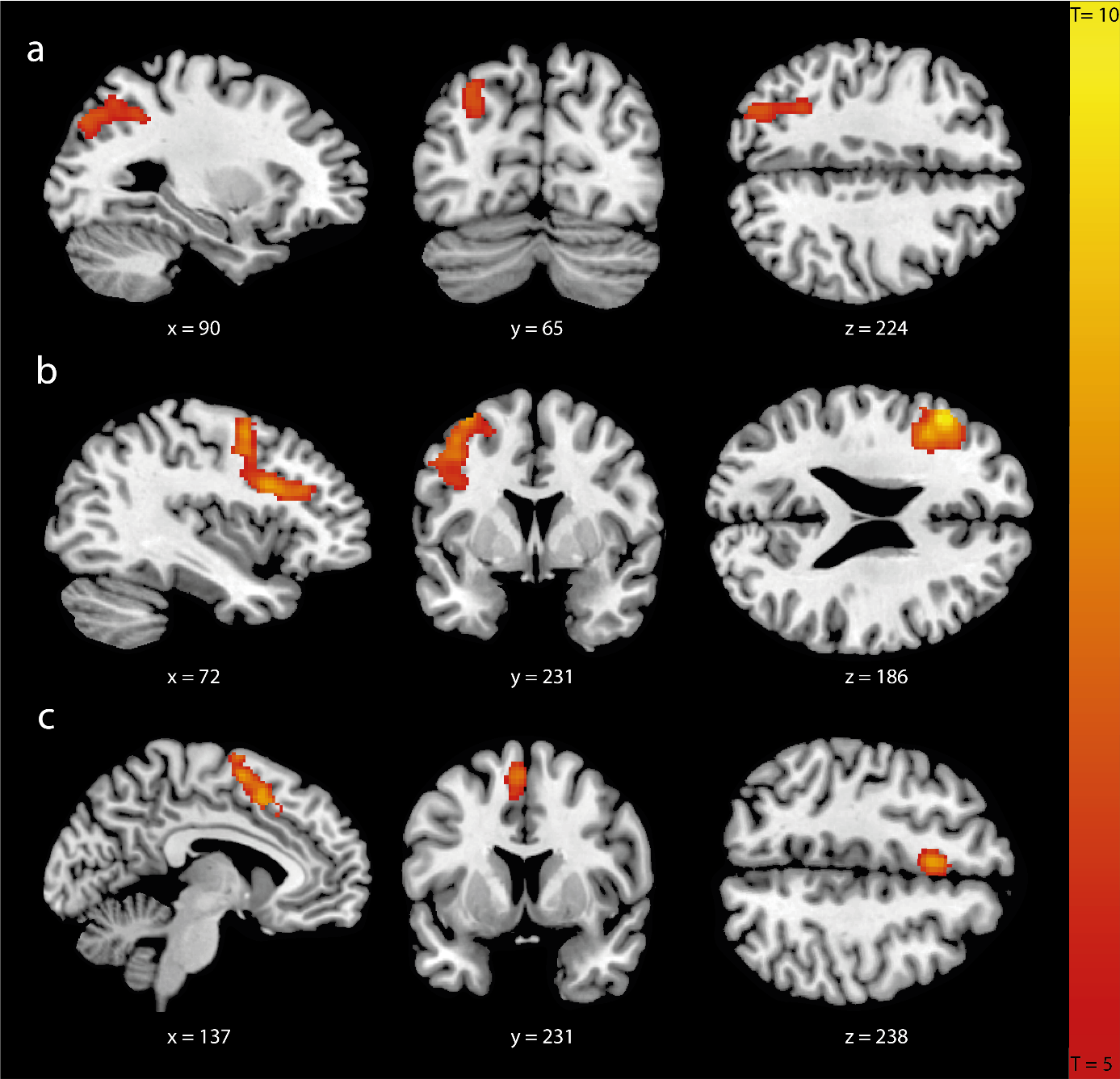


**Figure S3. Neural Stroop interference effect.** Contrast of incongruent versus congruent words (incongruent > congruent) in **a**) inferior parietal/middle occipital gyrus; **b**) inferior frontal gyrus; **c**) supplementary motor cortex. Images are shown in neurological convention (left=left) with sagittal, coronal and axial slice coordinates as defined in MNI152 space. For illustrative purposes full brain statistical parametric maps were thresholded at pFWE < 0.05, and corrected for age, sex and pre-surgery BMI.

**Table S2.** Linear regression models estimating the relation between brain activation during response inhibition and BMI.

|  | Beta Clusters | | |
| --- | --- | --- | --- |
|  | Inferior-parietal/Middle-occipital gyrus | Inferior-frontal gyrus | Supplementary motor cortex |
|  | β (95% CI) | β (95% CI) | β (95% CI) |
| Pre-surgery BMI |  |  |  |
| *Crude model* | -0.14 (-0.12 to 0.39) | 0.01 (-0.25 to 0.27) | -0.02 (0.28 to 0.24) |
| *Adjusted for age and sex* | 0.14 (-0.11 to 0.40) | 0.03 (-0.23 to 0.29) | -0.02 (0.27 to 0.24) |
| BMI 6 months |  |  |  |
| *Crude model* | 0.14 (-0.12 to 0.41) | 0.08 (-0.17 to 0.35) | -0.05 (-0.31 to 0.22) |
| *Adjusted for age and sex* | 0.15 (-0.12 to 0.42) | 0.10 (-0.16 to 0.37) | -0.05 (-0.31 to 0.22) |
| *Full model* | -0.02 (-0.19 to 0.14) | 0.06 (-0.10 to 0.22) | 0.01 (-0.17 to 0.15) |
| BMI 1 year |  |  |  |
| *Crude model* | 0.07 (-0.20 to 0.34) | 0.11 (-0.15 to 0.37) | -0.06 (-0.32 to 0.20) |
| *Adjusted for age and sex* | 0.07 (-0.20 to 0.35) | 0.12 (-0.15 to 0.38) | -0.05 (-0.31 to 0.21) |
| *Full model* | -0.00 (-0.19 to 0.19) | 0.08 (-0.10 to 0.26) | -0.05 (-0.23 to 0.13) |

*Beta-coefficients with 95% confidence intervals. In the full model we adjusted for age, sex and pre-surgery BMI.*

*Abbreviations: BMI = body mass index*


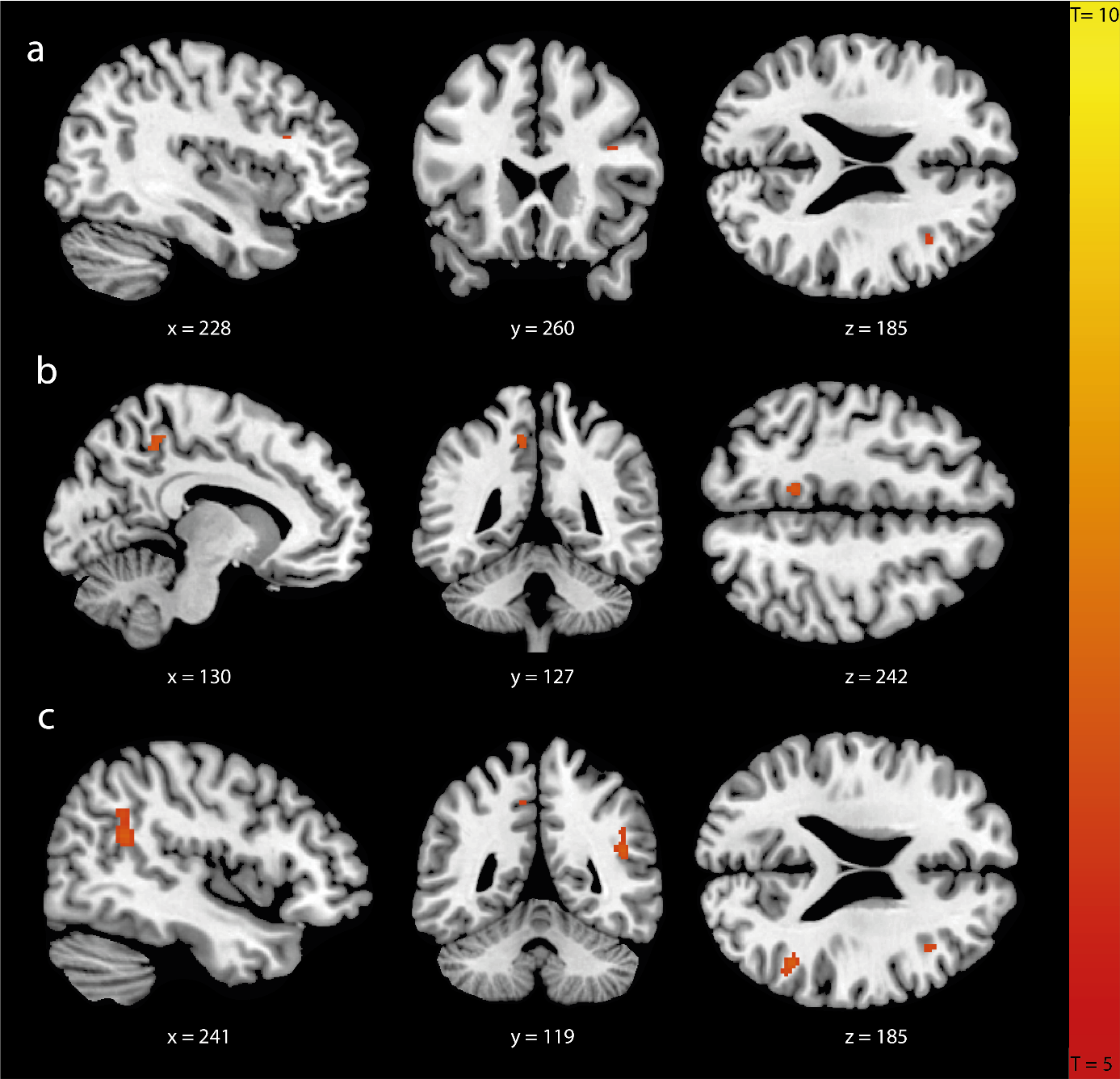


**Figure S4. Subthreshold changes in task-dependent connectivity**. Images are shown in neurological convention (left=left) with sagittal, coronal and axial slice coordinates as defined in MNI152 space. For illustrative purposes full brain statistical parametric maps were thresholded at pFWE(cluster)<0.001, uncorrected. Subthreshold cluster for the seed in **a**) inferior parietal/middle occipital gyrus (x = 38, y = 22, z = 22; p = 0.325; pFWE = 1.000); **b**) inferior frontal gyrus (x = -10, y = -46, z = 48; p = 0.049; pFWE = 0.992); **c**) supplementary motor cortex (x = 46, y = -50, z = 22; p = 0.003; pFWE = 0.368).

**References**

**1.** Esteban O, Markiewicz CJ, Blair RW, et al. Fmriprep: A robust preprocessing pipeline for functional mri. *Nat Methods.* 2019;16(1):111-116. doi: 10.1038/s41592-018-0235-4.

**2.** Gorgolewski K, Burns CD, Madison C, et al. Nipype: A flexible, lightweight and extensible neuroimaging data processing framework in python. *Front Neuroinform.* 2011;5:13. doi: 10.3389/fninf.2011.00013.

**3.** Tustison NJ, Avants BB, Cook PA, et al. N4itk: Improved n3 bias correction. *IEEE Trans Med Imaging.* 2010;29(6):1310-1320. doi: 10.1109/Tmi.2010.2046908.

**4.** Avants BB, Epstein CL, Grossman M, Gee JC. Symmetric diffeomorphic image registration with cross-correlation: Evaluating automated labeling of elderly and neurodegenerative brain. *Med Image Anal.* 2008;12(1):26-41. doi: 10.1016/j.media.2007.06.004.

**5.** Zhang YY, Brady M, Smith S. Segmentation of brain mr images through a hidden markov random field model and the expectation-maximization algorithm. *Ieee Transactions on Medical Imaging.* 2001;20(1):45-57. doi: 10.1109/42.906424.

**6.** Dale AM, Fischl B, Sereno MI. Cortical surface-based analysis - i. Segmentation and surface reconstruction. *Neuroimage.* 1999;9(2):179-194. doi: 10.1006/nimg.1998.0395.

**7.** Klein A, Ghosh SS, Bao FS, et al. Mindboggling morphometry of human brains. *PLoS Comput Biol.* 2017;13(2). doi: 10.1371/journal.pcbi.1005350.

**8.** Evans AC, Janke AL, Collins DL, Baillet S. Brain templates and atlases. *Neuroimage.* 2012;62(2):911-922. doi: 10.1016/j.neuroimage.2012.01.024.

**9.** Jenkinson M, Bannister P, Brady M, Smith S. Improved optimization for the robust and accurate linear registration and motion correction of brain images. *Neuroimage.* 2002;17(2):825-841. doi: 10.1006/nimg.2002.1132.

**10.** Cox RW, Hyde JS. Software tools for analysis and visualization of fmri data. *NMR Biomed.* 1997;10(4-5):171-178. doi: 10.1002/(Sici)1099-1492(199706/08)10:4/5<171::Aid-Nbm453>3.0.Co;2-L.

**11.** Posse S, Wiese S, Gembris D, et al. Enhancement of bold-contrast sensitivity by single-shot multi-echo functional mr imaging. *Magn Reson Med.* 1999;42(1):87-97. doi: 10.1002/(Sici)1522-2594(199907)42:1<87::Aid-Mrm13>3.0.Co;2-O.

**12.** Greve DN, Fischl B. Accurate and robust brain image alignment using boundary-based registration. *Neuroimage.* 2009;48(1):63-72. doi: 10.1016/j.neuroimage.2009.06.060.

**13.** Power JD, Mitra A, Laumann TO, Snyder AZ, Schlaggar BL, Petersen SE. Methods to detect, characterize, and remove motion artifact in resting state fmri. *Neuroimage.* 2014;84:320-341. doi: 10.1016/j.neuroimage.2013.08.048.

**14.** Behzadi Y, Restom K, Liau J, Liu TT. A component based noise correction method (compcor) for bold and perfusion based fmri. *Neuroimage.* 2007;37(1):90-101. doi: 10.1016/j.neuroimage.2007.04.042.

**15.** Satterthwaite TD, Elliott MA, Gerraty RT, et al. An improved framework for confound regression and filtering for control of motion artifact in the preprocessing of resting-state functional connectivity data. *Neuroimage.* 2013;64:240-256. doi: 10.1016/j.neuroimage.2012.08.052.

**16.** Patriat R, Reynolds RC, Birn RM. An improved model of motion-related signal changes in fmri. *Neuroimage.* 2017;144:74-82. doi: 10.1016/j.neuroimage.2016.08.051.

**17.** Pruim RHR, Mennes M, van Rooij D, Llera A, Buitelaar JK, Beckmann CF. Ica-aroma: A robust ica-based strategy for removing motion artifacts from fmri data. *Neuroimage.* 2015;112:267-277. doi: 10.1016/j.neuroimage.2015.02.064.

**18.** Lanczos C. Evaluation of noisy data. *JSTOR.* 1964;76-85. doi: 10.1137/0701007.
